# What is the appropriate treatment strategy for cryptogenic multifocal ulcerative stenosing enteritis? A single-center experience from China

**DOI:** 10.3389/fmed.2022.926800

**Published:** 2022-08-11

**Authors:** Pengguang Yan, Kemin Li, Yang Cao, Dong Wu, Ji Li, Jiaming Qian, Weixun Zhou, Jingnan Li

**Affiliations:** ^1^Key Laboratory of Gut Microbiota Translational Medicine Research, Department of Gastroenterology, Peking Union Medical College Hospital, Chinese Academy of Medical Sciences, Beijing, China; ^2^Peking Union Medical College, Beijing, China; ^3^Department of Pathology, Peking Union Medical College Hospital, Beijing, China

**Keywords:** CMUSE (cryptogenic multifocal ulcerous stenosing enteritis), steroids resistance, treatment strategy, *PLA2G4A*, *SLCO2A1*, prostaglandin pathway

## Abstract

**Background:**

There are few reports on standard treatment and long-term prognosis in patients with cryptogenic multifocal ulcerative stenosing enteritis (CMUSE), particularly in patients in whom remission could not be induced by steroids. The aim of this study was to evaluate the treatment response and progression-free periods of patients with CMUSE and to identify the factors predictive of steroid resistance.

**Methods:**

This was a retrospective cohort study that included 25 patients with clinically confirmed CMUSE between 1984 and 2021 from the enteropathy clinic of a tertiary care center. For statistical analyses, chi-square test or Fisher’s exact test were used for categorical variables. Survival curves were plotted using the Kaplan–Meier method.

**Results:**

The overall median progression-free period was 48 months (range, 1–108 months) after comprehensive therapy, and initial manifestation with severe bleeding rather than ileus was associated with the long-term efficacy. Patients with steroid resistance (*N* = 10, 55.6%) had poor prognosis, and non-responders had more favorable baseline clinical characteristics, with a higher percentage of female patients (60% vs. 12.5%), earlier disease onset (26.5 years vs. 39 years), rapid progression (42 vs. 108 months), severe anemia (80% vs. 50%), and hypoalbuminemia (50% vs. 0%), in accord with lymphangiectasia or angioectasia identified in pathology.

**Conclusion:**

There is no guaranteed treatment strategy in the maintenance of long-term clinical remission for CMUSE patients, particularly in whom with steroid resistance. Female patients with early symptoms onset, severe gastrointestinal hemorrhage and hypoalbuminemia seem to have poor long-term prognosis.

## Introduction

Cryptogenic multifocal ulcerative stenosing enteritis (CMUSE) is an extremely rare clinical condition that morphologically presents as multiple, shallow, and short mucosal ulcerations with focal circumferential fibrous strictures of the small intestine. It is clinically characterized by chronic and relapsing (sub) ileus episodes, intermittent obscure intestinal bleeding, and protein losing enteropathy (albeit not always) without a systematic inflammatory status except during the acute ileus (1, 2). Since the first case (3) of CMUSE reported in 1964, approximately 100 cases have been documented. However, owing to limited large-scale research, there is no standard diagnosis for CMUSE thus far (4–6), Clinical diagnosis is based on manifestations, typical endoscopic and radiologic features, and histopathological examinations. Before final diagnosis, infectious, ischemic, or non-steroidal anti-inflammatory drug (NSAID) enteropathy, Crohn’s disease, isolated vasculitis, and other autoimmune or small bowel malignancies should be excluded.

Even though the pathomechanism of CMUSE remains to be fully elucidated, the available evidence and research accumulated over the past decade recommends that this disease could be subtyped according to prostaglandin bioavailability. A loss-of- function mutation of the *PLA2G4A* gene downregulates the catalytic generation of a prostaglandin precursor, eicosanoids, leading to defective prostaglandin production (7). Furthermore, solute carrier organic anion transporter family member 2A1 (*SLCO2A1*) is another identified mutated gene in patients with CMUSE or a similar entity called chronic enteropathy associated with *SLCO2A1* gene (CEAS). Typically, the wild type of this gene encodes a transporter that imports prostaglandin to exert its intracellular signaling or for its further degradation; however, mutation of *SLCO2A1* impairs the intracellular utility of prostaglandin and causes its accumulation (8). However, not all patients with CMUSE have prostaglandin pathway impairment.

There have been few case reports on this subject, thus limiting the evidence for therapy response; however, glucocorticoids are considered effective in alleviating symptoms in most patients with CMUSE (6, 9). However, steroid-refractory CMUSE has been reported (10, 11), and these patients tended to have poor prognosis with the condition being refractory even after several surgeries. In a Korean CMUSE cohort (12), which included 12 patients who received corticosteroid treatment, the overall response rate was only of 25%. Besides, the experience with other immunomodulators, such as azathioprine, thalidomide, infliximab, and vedolizumab, is limited (4, 11, 13). Therefore, this study was conducted in a tertiary center in China to retrospectively evaluate the clinical phenotypes and prognosis of patients with CMUSE.

## Materials and methods

### Patients

We retrospectively enrolled patients who were clinically diagnosed as having CMUSE after appropriate inpatient investigations between August 1984 and March 2021 at Peking Union Medical College Hospital. After a thorough review of medical records by an expert in enteropathy, we included 12 patients with CMUSE pathologically confirmed by surgical resection and 13 patients highly suspected as having CMUSE based on typical radiology features and mucosal biopsy pathology. The reference diagnostic criteria were as follows: (1) refractory and occult blood loss from the gastrointestinal (GI) tract; (2) unexplained scattering and short strictures of the small intestine; (3) superficial ulcers affecting the mucosa and submucosa; (4) no signs of systematic inflammation except during acute ileus; and (5) the absence of a relatively more common etiology of small intestine ulcerative disease (e.g., NSAID enteropathy, Crohn’s disease, celiac disease, and small bowel malignancies).

Demographics and disease characteristics, such as symptoms, disease duration, medication history of taking NSAIDs, pathological features (e.g., submucosal fibrosis, angioectasia and lymphangiectasis), and laboratory, radiographic, and endoscopic findings (e.g., location and morphology of ulcer and strictures and penetrating disease features), were collected. Data on clinical manifestation and CT/MR enterography response after induction and maintenance with specific medications (e.g., mesalazine, corticosteroids, azathioprine, methotrexate, and thalidomide) during follow-up were obtained from electronic medical files and call visits. All patients were followed up for at least 6 months. Clinical improvement was defined as follows: (1) HGB sustained ≥ 90 g/L without repeated blood transfusions, (2) ≥ 50% reduction in numeric pain rating scale scores, and (3) no emergency visits for episodes of ileus and obstruction within 6 months postoperatively. Clinical remission was defined as abdominal pain relief and HGB sustained ≥ 120 g/L without iron supplementation. If CTE or MRE was performed after induction, radiological remission was defined as normalization of bowel wall without thickness or hyperenhancement patterns.

For categorical variables, the chi-square test or Fisher’s exact test was used when appropriate to compare baseline characteristics between steroid responders and non-responders. Progression-free period was the time from treatment to deterioration of symptoms or relapse compared with clinical improvement. The most recent follow-up data included were of August 31, 2021. Survival curves were plotted using the Kaplan– Meier method. *P* < 0.05 indicated a statistically significant difference. Statistical analysis was performed with SPSS 22 (SPSS, Inc., Chicago, IL, United States).

## Results

### Patient characteristics

**FIGURE 1:**
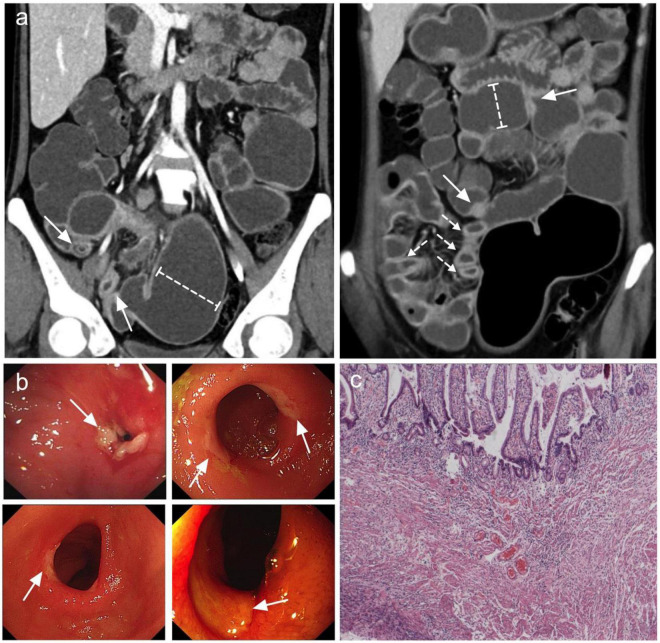
Most common features observed in CMUSE. **(a)** Coronal CT enterography images demonstrate multiple short strictures, with mild stratified hyperenhancement (arrows) and proximal lumen dilation (dashed-capped lines). **(b)** Double balloon enteroscopy demonstrates circumferential shallow ulcers in ileum or jejunum (arrows). **(c)** HE stain of the ileum demonstrates superficial ulcers involving mucosa and submucosa with angioectasia and congestion.

A total of 25 patients (14 men and 11 women) were included in the analysis (Table 1). The median age at the time of diagnosis was 37 years (range, 18–61 years), and the median interval from the onset of initial manifestation to final diagnosis was 96 months (range, 5–324 months). Occult GI bleeding (22/25, 88%) and abdominal pain (22/25, 88%) were the most common symptoms. Among the bleeding patients, 17 patients had overt melena or hematochezia with minimum hemoglobin level ≤ 60 g/L. In addition, 15 patients experienced relapsing episodes of sub-ileus. The median serum albumin level was 33 g/L (range, 11–47 g/L), and 5 patients had severe hypo-albuminemia with minimum serum albumin level ≤ 20 g/L. The most common site of small intestine ulcers was the ileum (Figure 1a), with all patients (25/25, 100%) showing ileal involvement; 10 of these patients had concomitant jejunal or duodenal lesions, and only 4 patients had coexisting terminal ileal involvement. Superficial ulcers and strictures were the typical pathological features of CMUSE (Figure 1b), and submucosa fibrosis (15/25, 60%) and lymphangiectasia/angioectasia (11/25, 44%) could be observed under mucosal ulcers (Figure 1c). However, only 4/25 (16%) of the patients with CMUSE had aberrant crypt foci, which is the classic pathologic finding of inflammatory bowel diseases.

**TABLE 1:** Baseline and clinical characteristics of patients with CMUSE.

Baseline characteristics	All patients (*N* = 25)
Male	56%
Age at diagnosis (years) < 40	60%
Disease duration before diagnosis (years) ≥ 8	52%
**Clinical manifestations**	
Abdominal pain	88%
Ileus	56%
Hematochezia	12%
Melena	84%
Capsule retention	75% (*N* = 12)
**Laboratory findings**	
HGBmin ≤ 60 g/L	64%
Albmin ≤ 20 g/L	20%
CRP ≤ 10 mg/L	80%
ESR ≤ 20 mm/h	92%
**Location of ulcer and strictures**	
Duodenum	24%
Jejunum	40%
Ileum	100%
Terminal ileum	16%
**Pathologic findings**	
Submucosa fibrosis	60%
Lymphangiectasia or angioectasia	44%
Aberrant crypt foci	16%

### Treatment response

**FIGURE 2:**
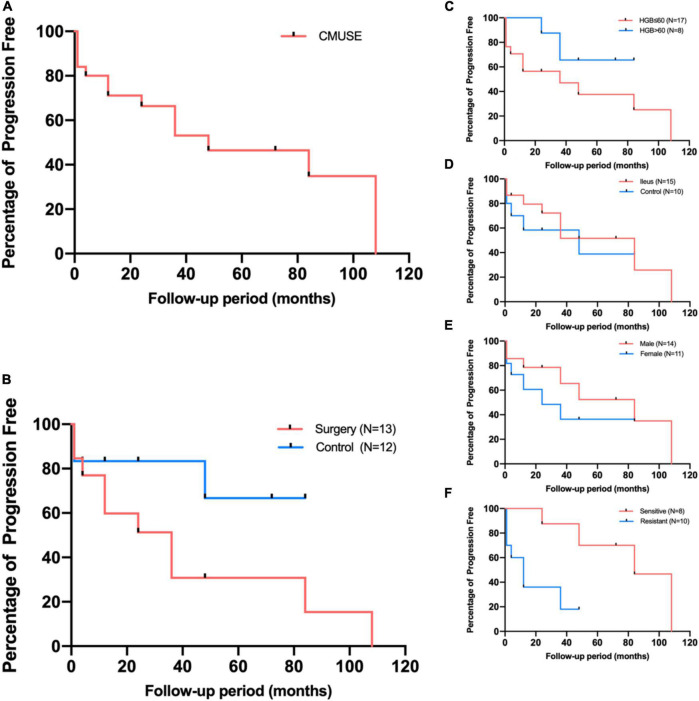
Kaplan-Meier estimates for patients free from progression **(A)**, and subgroup analyses according to surgery **(B)**, presenting manifestations **(C,D)**, gender **(E)**, and corticosteroids response **(F)**.

The overall median follow-up duration was 47 months (range, 6–146 months); 13 patients accepted surgical (5 for capsule retention, 6 for ileus and bleeding, and 2 for ileus only) and further medical maintenance remission therapy, and the remaining 12 patients received only medical induction and maintenance therapy. All patients were followed up in outpatient department or call visits to assess symptom changes, 9 patients received repeated enteroscopy or enterography to assess the improvement of mucosal lesions. After comprehensive treatment, 14 patients showed clinical improvement, among whom 8 patients showed clinical remission. However, only three patients achieved radiographic improvement. Although mesalazine was mostly the preliminary treatment option, only 6/21 patients (28.6%) administered mesalazine showed clinical improvement, and the improvement mostly did not persist, suggesting that it had limited clinical benefit. Glucocorticoids were regarded as the first-line therapy, 18 patients received standard induction of remission with systematic corticosteroids (0.6–1.0 mg/kg/d prednisone or equivalents according to the degree of mucosal enhancement on radiology) for at least 4 weeks followed by gradually decrement if signs of clinical improvement were observed. However, 10 patients (55.6%) did not show clinical benefit (Table 2); most of the non-responders (7/10, 70%) had refractory occult blood loss, and the remaining non-responders had persistent abdominal pain or episodes of sub-ileus. With respect to corticosteroid treatment, compared to the responders, the non-responders had more favorable baseline clinical characteristics, with a higher percentage of female patients, younger age at diagnosis, and a marginally shorter disease duration. Besides, the non-responders tended to have a higher percentage of severe anemia and hypoalbuminemia, in accord with more frequent lymphangiectasia or angioectasia in pathology; however, the differences were not statistically significant (Table 3). Notably, 1/5 (20%), 1/4 (25%), and 5/11(45.5%) patients who received azathioprine, methotrexate, and thalidomide, respectively, achieved clinical remission. Kaplan–Meier analysis revealed the median progression- free period as 48 months (range, 1–108 months) under comprehensive treatment (Figure 2A). Subgroup analysis found that postoperative relapse occurred in 10 out of 13 patients (76.9%) who received surgical bowel resection; the median progression-free period was 36 months (range, 1–108 months) (Figure 2B). Patients who presented with overt blood loss from the GI tract with HGB ≤ 60 g/L had poor prognosis (Figure 2C). Early recurrence was observed in this subgroup, with less than 12 months of 60% progression-free period compared with > 84 months in patients with HGB > 60 g/L (*P* = 0.08). Gender and ileus were not significant prognostic factors for the progression- free period in patients with CMUSE (Figures 2D,E). However, patients with glucocorticoid resistance had worse prognosis (*P* < 0.05) despite being administered various immunosuppressors as combination treatment (Figure 2F).

**TABLE 2:** The overall treatment efficacy in patients with CMUSE and differences between steroids-response subtypes.

	All patients (*N* = 25)	Steroid responsive (*N* = 8)	Steroid resistant (*N* = 10)	*P*-values
**Reasons for surgery**	(*N* = 13)	(*N* = 3)	(*N* = 7)	
Ileus	61.5%	66.7%	57.1%	1
GI bleeding	46.2%	66.7%	42.9%	1
Capsule retention	38.5%	33.3%	42.9%	1
**Effectiveness of medical maintenance therapy**				
Mesalazine	28.6% (*N* = 21)	14.3% (*N* = 7)	12.5% (*N* = 8)	1
Glucocorticoids	44.4% (*N* = 18)	–	–	–
Azathioprine	20% (*N* = 5)	100% (*N* = 1)	0% (*N* = 4)	0.2
Methotrexate	25% (*N* = 4)	100% (*N* = 1)	0% (*N* = 3)	0.25
Thalidomide	45.5% (*N* = 11)	100% (*N* = 2)	14.3% (*N* = 7)	0.083

**TABLE 3:** The differences of baseline, and clinical characteristics in CMUSE between steroids-response subtypes.

Baseline characteristics	Steroid responsive (*N* = 8)	Steroid resistant (*N* = 10)	*P*-values
Male	87.5%	40%	0.066
Age at diagnosis (years) < 40	25%	80%	0.054
Disease duration before diagnosis (years) ≥ 8	62.5%	40%	0.637
**Clinical manifestations**			
Abdominal pain	100%	80%	0.477
Ileus	62.5%	40%	0.637
Hematochezia	12.5%	0%	0.444
Melena	87.5%	100%	0.444
**Laboratory findings**			
HGBmin ≤ 60 g/L	50%	80%	0.321
Albmin ≤ 20 g/L	0%	50%	0.036
CRP ≤ 10 mg/L	100%	70%	0.216
ESR ≤ 20 mm/h	100%	80%	0.477
**Location of ulcer and strictures**			
Duodenum	25%	30%	1
Jejunum	25%	50%	0.367
Ileum	100%	100%	1
Terminal ileum	25%	10%	0.559
**Pathologic findings**			
Submucosa fibrosis	37.5%	70%	0.342
Lymphangiectasia or angioectasia	25%	80%	0.054
Aberrant crypt foci	0%	20%	0.477

## Discussion

In this study, we reported the clinical characteristics and long-term treatment outcomes of CMUSE in our cohort. To our knowledge, this population is the largest cohort thus far to undergo a retrospective evaluation of treatment response and risk factors of treatment failure. Owing to the rarity and inconsistency in the presentation of CMUSE, conducting prospective controlled trials to elucidate optimal treatment strategies is difficult. Therefore, most reports on this subject have been case reports or case series. In our cohort, most cases showed disease onset at a young age and presented with abdominal pain (88%) and intermittent bouts of melena (84%), with a median disease duration of 8 years before the final diagnosis. Occult blood loss from the GI was more prominent in this cohort; 64% of these patients had a history of severe iron- deficiency anemia, which required repeated blood transfusions and regular iron supplementation orally. Interestingly, bleeding rather than ileus was associated with the long-term efficacy of comprehensive therapy. The most common events responsible for the failure of corticosteroid induction were early bouts of melena. There were distinctive characteristic differences between corticosteroid responders and non-responders. Here, 20–40-year-old women having severe iron-deficiency anemia were more likely to exhibit steroid resistance. The non-responders usually had earlier disease onset and shorter intervals between the onset of initial manifestation and final diagnosis; however, submucosa fibrosis, which was considered as the marker of late phase (4), was more prevalent in this population. These data indicated that corticosteroid-resistant CMUSE patients had rapid disease progression and poor long-term prognoses.

Dating back to the discovery of CMUSE, corticosteroids were regarded as the first- line therapy with respectable response, and in several case reports, the efficacy of corticosteroids was included in the diagnostic criteria (6, 12, 13). However, few case reports have discussed steroid-refractory CMUSE patients (10, 11, 13–15), and upon scrutiny, similar cases were observed in a large cohort as well (4, 16). Generally, these patients had severe iron-deficiency anemia and hypoalbuminemia (10, 11, 17), and poor steroid response seemed to be more common in female patients (10, 11, 16). Recently, loss-of-function mutations in the *SLCO2A1* gene was identified in patients with superficial small bowel ulcers and short strictures, presenting with chronic or intermittent bouts of melena, protein loss, and abdominal pain. Some researchers defined these patients as having a new condition called chronic enteropathy associated with the *SLCO2A1* gene (CEAS) rather than classifying them under a subtype of CMUSE. Although CEAS predominantly occurred in women and had poor corticosteroid response, it had identical clinical characteristics and imaging, endoscopic, and histological features to CMUSE (8, 18). Furthermore, CEAS and CMUSE did not differ in terms of the segment involved, with the ileum being the common site of involvement. We did acknowledge that owing to the lack of genetic analysis, *SLCO2A1* mutations may have been underestimated in previous published works (19). In our cohort, genetic sequencing was performed for 5 out of 10 patients with corticosteroid resistance; consequently, 3 were found to harbor mutant *SLCO2A1* alleles and 1 carried a mutation in the *PLA2G4A* gene. Further genetic sequencing could help differentiate and establish CMUSE subtypes.

Thus far, there is limited information on treatment experience for steroid- resistant/refractory CMUSE, and there is a risk for selection bias as cases with a positive outcome are more likely to be reported. Immunosuppressors were administered in several case reports of steroid-refractory CMUSE patients (4, 11, 13); however, the results were disappointing. In our cohort, four and three steroid-refractory patients received maintenance treatment with azathioprine and methotrexate, respectively; however, there were no clinical benefits. Given the potential effect of thalidomide on angiogenesis and vascular permeability inhibition, it can effectively prevent severe bleeding in Crohn’s disease (20, 21). Conversely, clinical remission was achieved in only 1/7 patients who received rescue therapy of thalidomide after early rebleeding following corticosteroid induction, with the remaining patients showing poor prognosis with continuous enteral nutrition support, repeated blood transfusions, and intermittent symptomatic treatments. Biologic therapy was attempted in steroid-refractory CMUSE patients and showed encouraging preliminary results; however, its long-term efficacy needs to be checked with further follow-up data (4, 15). Surgery was required in 70% of patients with steroid- refractory CMUSE, despite which disease recurrence remained common in this population (4, 16). Notably, the postoperative progression-free period was shorter in these patients than in those who achieved remission through medication only. This finding could be partially attributed to lack of medical maintenance of remission or to relatively inadequate treatment intensity by mesalazine postoperatively. Therefore, alternative treatment strategies are of substantial clinical importance.

Recently, potential therapy targets have been unearthed owing to the increasing understanding of the etiology of CMUSE. Prostaglandin metabolic pathways and functioning participate in the pathogenesis of the enteropathy. Patients with CMUSE have been identified to harbor a mutation in *PLA2G4A*, which encodes a calcium- activated phospholipase that regulates the biosynthesis of eicosanoids by liberating arachidonic acid from phospholipids (7, 22). In patients with the *PLA2G4A* mutation, the levels of prostaglandins and thromboxanes are extremely low due to the impaired precursor production. These prostaglandins participate in maintaining mucosal blood flow, regulating mucosal immunity by inhibiting mast cell activation and leukocyte chemotaxis, and establishing mucous barrier by stimulating mucus and bicarbonate secretion. The intestinal lesions in individuals with the loss-of-function mutation of *PLA2G4A* resemble those in NSAID-induced chronic enteropathy, wherein COX inhibition by NSAIDs impairs prostaglandin biosynthesis (12, 23). Besides, platelet dysfunction has been observed in the mutation phenotype, which is similar to an aspirin-like antiplatelet effect. Although platelet aggregation is diminished by lack of thromboxanes, the dysfunction can reportedly be reversed by exogenous arachidonic acid supplementation (7). In patients with *PLA2G4A* mutation who presented with persistent occult GI blood loss, misoprostol, a synthetic prostaglandin E1, has been used for supplementary therapy with relatively satisfying efficacy; however, the bleeding diathesis is not completely reversed with bleeding recurrence noted during the long- term follow-up.

*SLCO2A1* is another identified mutated gene in CMUSE patients. It encodes a prostaglandin transporter that uptakes prostaglandin into tissues for further intracellular signaling or degradation. Unlike patients with *PLA2G4A* mutation, patients with *SLCO2A1* mutation show a globally elevated level of circulating prostaglandins. Notably, prostaglandins are potent inflammatory mediators that induce inflammasome activation in macrophages. Susceptibility of *SLCO2A1*-deficient mice to DSS-induced colitis has been reported, and when macrophages exhibit increased secretion of prostaglandin E2 (PGE2) which could not be imported for further degradation, extracellular prostaglandin signaling gets activated, which consequently leads to inflammasome activation in macrophages (24, 25). Conversely, prostaglandins promote intestinal mucus and fluid secretion through cell membrane receptors, which is followed by second messenger activation and further signal transduction (26). However, the protective effect of PGE2 on injured mucosal epithelium does not compensate for the proinflammatory effect of excessive PGE2 on immune homeostasis. Besides, PGE2 could exert its anti-fibrotic effect in response to local inflammation, including the inhibition of fibroblast proliferation and migration (27, 28). Theoretically, excessive PGE2 appears to ameliorate intestinal fibrosis in *SLCO2A1*-deficient mice; however, aberrant disposition of PGE2 was observed in these mice (higher concentrations in the lumen and lower tissue levels), thus leading to severe fibrotic tendencies in organ tissues (29). Furthermore, the inhibition of endothelial cell prostaglandin extraction and degradation caused by the loss-of-function mutation of *SLCO2A1* reinforces the vasodilatory effect of PGE2 (30), which may be responsible for the prominent pathologic findings of lymphangiectasia or angioectasia in parts of our patients with CMUSE. Therefore, prostaglandin metabolic pathways may be a therapeutic target to restrain excessive fibrosis and reduce bleeding diathesis. Cyclooxygenase-2 (COX-2) inhibitor has been administered to patients with the loss-of-function mutation of *SLCO2A1* presenting with primary hypertrophic osteoarthropathy (31); however, patients with GI symptoms are not responsive to this treatment (32).

This present study has some limitations. This was a retrospective study, and it had a limited number of patients from a single tertiary center. There are chances of biases from investigators in terms of the initial evaluation and treatment options from clinicians, which may mix with cofounding factors that could affect drug efficacy assessment. Besides, upon evaluating treatment efficacy solely on the basis of clinical parameters, few patients were found to achieve evident radiological improvement. Considering the rarity of CMUSE and lack of literature in this regard, the sample size is sufficient to assess the efficacy of the integrated treatment; however, statistical significance was not reached with most potential risk factors. Besides, not all patients underwent genetic analysis or specific prostaglandin metabolite detection to further differentiate whether there was functional impairment in prostaglandin metabolic pathways.

Taken together, the overall treatment response and progression-free period in CMUSE patients is frustrating, particularly in patients with steroid-refractory CMUSE. Female patients presenting with severe bleeding diathesis and protein loss with early disease onset and rapid progression have extremely poor long-term prognosis. Future investigations on the efficacy of prostaglandin pathway mediation according to physiopathologic mechanisms are warranted.

## Data availability statement

The original contributions presented in this study are included in the article/supplementary material, further inquiries can be directed to the corresponding author/s.

## Ethics statement

The studies involving human participants were reviewed and approved by the Ethics Committee of Peking Union Medical College Hospital. Written informed consent for participation was not required for this study in accordance with the national legislation and the institutional requirements.

## Author contributions

PY and JNL designed the study and reviewed all the medical records of the potential patients included and enrolled patients. KL and YC wrote the statistical plan. KL and PY followed up patients. WZ reviewed pathological sections. PY drafted the manuscript. DW, JL, and JQ critically revised the manuscript. All authors approved the final version of manuscript.
